# Exclusion of elective nodal irradiation is associated with minimal elective nodal failure in non-small cell lung cancer

**DOI:** 10.1186/1748-717X-4-5

**Published:** 2009-01-30

**Authors:** Erik P Sulman, Ritsuko Komaki, Ann H Klopp, James D Cox, Joe Y Chang

**Affiliations:** 1 Department of Radiation Oncology, the University of Texas M. D. Anderson Cancer Center, 1515 Holcombe Boulevard, Houston, TX, USA

## Abstract

**Background:**

Controversy still exists regarding the long-term outcome of patients whose uninvolved lymph node stations are not prophylactically irradiated for non-small cell lung cancer (NSCLC) treated with definitive radiotherapy. To determine the frequency of elective nodal failure (ENF) and in-field failure (IFF), we examined a large cohort of patients with NSCLC staged with positron emission tomography (PET)/computed tomography (CT) and treated with 3-dimensional conformal radiotherapy (3D-CRT) that excluded uninvolved lymph node stations.

**Methods:**

We retrospectively reviewed the records of 115 patients with non-small cell lung cancer treated at our institution with definitive radiation therapy with or without concurrent chemotherapy (CHT). All patients were treated with 3D-CRT, including nodal regions determined by CT or PET to be disease involved. Concurrent platinum-based CHT was administered for locally advanced disease. Patients were analyzed in follow-up for survival, local regional recurrence, and distant metastases (DM).

**Results:**

The median follow-up time was 18 months (3 to 44 months) among all patients and 27 months (6 to 44 months) among survivors. The median overall survival, 2-year actuarial overall survival and disease-free survival were 19 months, 38%, and 28%, respectively. The majority of patients died from DM, the overall rate of which was 36%. Of the 31 patients with local regional failure, 26 (22.6%) had IFF, 5 (4.3%) had ENF and 2 (1.7%) had isolated ENF. For 88 patients with stage IIIA/B, the frequencies of IFF, any ENF, isolated ENF, and DM were 23 (26%), 3 (9%), 1 (1.1%) and 36 (40.9%), respectively. The comparable rates for the 22 patients with early stage node-negative disease (stage IA/IB) were 3 (13.6%), 1(4.5%), 0 (0%), and 5 (22.7%), respectively.

**Conclusion:**

We observed only a 4.3% recurrence of any ENF and a 1.7% recurrence of isolated ENF in patients with NSCLC treated with definitive 3D-CRT without prophylactic irradiation of uninvolved lymph node stations. Thus, distant metastasis and IFF remain the primary causes of treatment failure and cancer death in such patients, suggesting little value of ENI in this cohort.

## Background

In recent decades, it has been standard practice when treating non-small cell lung carcinoma (NSCLC) to deliver 40 to 50 Gy to the uninvolved regional lymph nodes. Known as elective nodal irradiation (ENI), this therapy is targeted to ipsilateral and contralateral hilar/mediastinal nodes and occasionally to supraclavicular areas, with an additional 20 Gy delivered to the primary gross tumor through reduced fields [1].

In the era of 2-dimensional radiotherapy before the advent of positron emission tomography (PET) scanning for staging and before the use of adjunctive chemotherapy (CHT), Radiation Therapy Oncology Group (RTOG) studies had shown a potential survival benefit from ENI in stage III NSCLC [1]. The staging of regional lymph node involvement has been greatly enhanced with the help of PET. The addition of PET to clinical mediastinal staging with CT has improved sensitivity and specificity compared with CT alone. More accurate clinical staging with PET allows the radiation oncologist to include involved hilar and mediastinal nodes that were not visualized on the CT scan and thereby reduce the probability of elective nodal failure (ENF) [2]. Therefore, the concept of ENI has been challenged with recent data showing very low rates of elective nodal failure (ENF) using only involved-field radiation therapy (IFRT) without ENI in the era of computed tomography (CT)-based 3-dimensional conformal radiotherapy (3D-CRT) [3].

However, these results have been met with caution, and a recent survey reported that only 26% of the members of the American Society of Therapeutic Radiation Oncology treat NSCLC without ENI [4]. The most recent analysis of the pertinent literature conducted by the International Atomic Energy Agency concluded that the indication for ENI in such cases is uncertain [5]. It appears that more studies are needed to address this issue.

In our large single-institution study, we report the clinical outcome and pattern of failure of 3D-CRT without ENI in treating NSCLC after staging with PET scanning.

## Methods

### Patient selection and cancer staging

We retrospectively reviewed the clinical records of all consecutive patients who began their external beam radiation therapy for NSCLC during 2002 at The University of Texas M. D. Anderson Cancer Center. Patients were included for analysis if they were diagnosed with histologically or cytologically proven NSCLC not previously treated, received > 50 Gy definitive radiation to the primary tumor, had tumors stage I to IIIB, had pre- and post-treatment CT images available for review, and had complete radiation treatment records. Patients who had multiple lobes in the lung treated simultaneously were included based on the assumption that their tumors consisted of synchronous primary tumors. Disease was staged using chest CT and brain magnetic resonance imaging (MRI) in all patients. PET was also used for staging 75% of patients. Several patients were treated on clinical protocols involving induction or concurrent CHT. The majority of patients with locally advanced disease received concurrent platinum-based CHT along with their radiation therapy. This study was conducted with the approval of the Institutional Review Board (study RCR05-0263).

### Radiation treatment

Patients were treated using 3D conformal techniques planned using CT-based Pinnacle planning software (Philips Medical System, Andover, MA). Patients were treated in the supine position with their arms raised above their heads and were immobilized using a custom-made Vac-Lok cradle (Medtec, Orange City, IA). Gross tumor volume (GTV) was delineated based on the assessment of abnormalities in the CT images. We focused on the lung window to assess lung parenchymal disease and on the mediastinal window to assess lymph node and mediastinal involvement. When PET or PET/CT images were available, metabolically active lymph nodes were included in the GTV even if they appeared grossly normal on CT. Likewise, involved nodal regions diagnosed by histologic evaluation of biopsies taken during mediastinoscopy were also included in the GTV. Suspicious lymph nodes (> 1 cm in the shortest dimension) were included as well.

Clinical target volume was defined as the GTV plus an 8-mm margin to account for microscopic disease. First-echelon lymph nodes were included in the clinical target volume in some cases, if they were visible and adjacent to the gross disease and if coverage of these nodes did not elevate normal tissue toxicity significantly, as decided on an individual basis by the radiation oncologist. For the right mid lobe or right lower lobe, left lingular, or left lower lobe lesion, if a mediastinal lymph node was involved, the ipsilateral hilar and subcarinal lymph nodes were treated to 45–50 Gy. For a left upper lobe lesion, the aorto-pulmonary window lymph node were treated to 45–50 Gy if there was mediastinal lymph node involvement. Otherwise uninvolved elective nodal regions that might previously have been considered eligible for prophylactic treatment were excluded. Tumor motion was evaluated based on location, clinical judgment, and x-ray fluoroscopy. Another 10- to 15-mm margin was added to the planning target volume to take tumor motion and set-up uncertainty into consideration. Correction was made for tissue inhomogeneity in all treatment plans. Treatment was delivered using 6-MV or higher X-rays from a linear accelerator and typically consisted of three or four treatment fields, depending on the location of the tumor.

### Clinical endpoints

Clinical endpoints examined included overall survival, disease-free survival, locoregional recurrence, and distant metastasis (DM). At follow-up, patients were given chest CT scans every 3 months for 2 years and then every 6 months for 3 years. PET scans were commonly used to evaluate treatment response 3 to 6 months after the completion of radiotherapy and/or CHT. Clinical responses were evaluated using Response Evaluation Criteria in Solid Tumors (RECIST) based on both PET and CT images [6]. Local tumor recurrence was defined as progressive abnormal CT images corresponding to avid lesions on PET images obtained during routine patient follow-up. When a failure was noted in the clinical record, all imaging prior to that time was carefully reviewed and the date of failure coded based on the date the recurrence was first visible radiographically. Locoregional recurrences were classified as IFF if the majority of the recurrent tumor volume or an obvious origin of tumor recurrence was located within the radiation treatment field PTV. Locoregional failures were coded even if they occurred subsequent to DM. ENF was defined as any recurrence outside the treatment volume but located within bilateral hila, mediastinal lymph node regions, and bilateral supraclavicular areas (for upper lobe lesions). Isolated ENF was considered only in the absence of IFF and DM.

In cases of ENF, the site of recurrence was superimposed on the original treatment planning CT images and a recurrent tumor volume approximated. The mean dose received by this volume during the original treatment was determined using the treatment planning software to estimate dose to the nodal region that experienced recurrence. IFFs were presumed to have received the fully prescribed treatment dose.

### Statistics

Durations of survival and time-to-failure were determined from the date of pathologic diagnosis, and survival probabilities were determined by the Kaplan-Meier method using JMP 6.0.3 for Macintosh (SAS Institute, Cary, NC). Patients for whom the cause of death was unknown were coded as dead of disease. Timing of recurrence was scored at the time of the first image (PET and/or CT) that showed abnormalities.

## Results

### Patient population and treatment

Table 1 shows the characteristics of 115 consecutive patients with stage I to III NSCLC treated with 3D-CRT in 2002 at our institution (Table 1). The median follow-up time was 18 months (range, 3 to 49 months). The majority of patients had good performance status, with 89% categorized as either ECOG 0 or 1. Eighty-eight (77%) patients presented with locally advanced (stage III) disease and 80 (91%) patients received concurrent CHT as part of their treatment. Forty-four (38%) patients participated in prospective clinical trials. Radiation doses ranged from 58 to 69.6 Gy, with the majority of patients treated with either 63 Gy in 35 or 60 Gy in 30 daily fractions with concurrent CHT for stage III disease, 66 Gy in 33 daily fractions without CHT for stage 1 disease, or 69.6 Gy given as 1.2 Gy/fraction twice a day with concurrent CHT for stage III disease in the superior sulcus location.

**Table 1:** Patient characteristics (N = 115)

Median age, years (range)	65 (35 – 92)
Median follow-up, months (range)	18 (3 – 49)
Sex (%)	
male	58 (50)
female	57 (50)
ECOG performance status (%)	
0	31 (27)
1	71 (62)
2	8 (7)
3	1 (1)
Unknown	4 (3)
Weight loss before treatment (%)	
≥ 10%	24 (21)
< 10%	91 (79)
AJCC stage grouping (%)	
IA/IB	22 (19)
IIA/IIB	5 (4)
IIIA	34 (30)
IIIB	54 (47)
Comorbidities (%)	
COPD	30 (26)
Cardiovascular disease	59 (52)
Smoker	100 (87)
Pathologic classification (%)	
Adenocarcinoma	43 (37)
Squamous cell carcinoma	39 (34)
Large cell carcinoma	3 (3)
Mixed	2 (2)
Non-small cell not otherwise specified	28 (24)
Grade (%)	
Well-differentiated	7 (6)
Moderately differentiated	12 (10)
Poorly differentiated	55 (48)
No grade indicated	41 (36)
Location (%)	
Right	63 (54)
Left	49 (43)
Mediastinal nodal disease only	3 (3)
Staging workup (%)	
PET scan performed	86 (75)
Mediastinoscopy performed	16 (14)
Chemotherapy (%)	
Induction	55 (47)
Concurrent	
Taxane/platinum based	62 (54)
Other	18 (16)
Treated on clinical protocol (%)	44 (38)
Radiation treatment (%)	
Mean dose	64 Gy
> 66 Gy	8 (7)
> 63 – 66 Gy	29 (25)
60 – 63 Gy	75 (65)
< 60 Gy	3 (3)
Mean treatment duration (days)	45

A total of 115 patients (58 men and 57 women) were analyzed in this study, including 27 stage I/II and 88 stage IIIA/B cases. The median age at diagnosis was 65 years (range, 35 to 92). As noted, 75% of patients underwent PET for staging. Also, 70% of all patients and 85% of patients with stage IIIA/B disease received concurrent, platinum-based CHT.

### Clinical outcome and pattern of failure

**Figure 1:**
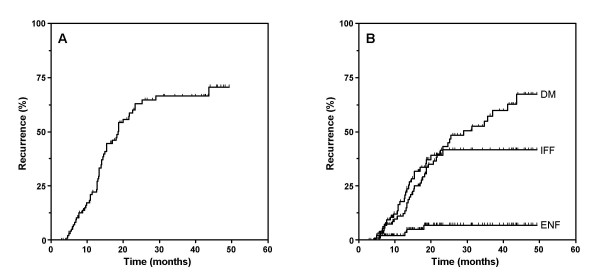
**Pattern of failure**. A. Probability of any disease recurrence by Kaplan-Meier method. B. Pattern of Failure. Distant metastasis (DM) remains the most common site of disease failure, followed by IFF and any ENF (p < 0.0001, log-rank test). Five of 115 patients developed ENF and only 2 of 5 ENFs were isolated ENFs.

The median follow-up time was 18 months (3 to 44 months) among all patients and 27 months (6 to 44 months) among survivors. The median overall survival duration and 2-year actuarial overall survival and disease-free survival percentages were 19 months, 38%, and 28%, respectively (Figure 1 and Table 2).

**Table 2:** Patient outcomes (N = 115).

Overall survival*	
Median (months)	19
1-yr (%)	72
2-yr (%)	38
Distant metastasis-free survival*	
Median (months)	35
1-yr (%)	90
2-yr (%)	60
Local control*	
Median (months)	not reached
1-yr (%)	83
2-yr (%)	60
Disease-free survival*	
Median (months)	14
1-yr (%)	63
2-yr (%)	28

* Kaplan-Meier method.

**Figure 2:**
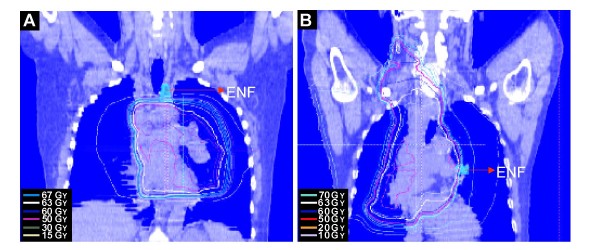
**Representative coronal reconstructions from planning CT images demonstrating locations of ENF**. The green contour represents ENF. The approximate location of the recurrences based on diagnostic CT imaging performed at the time of the recurrence is indicated (shaded area). A. Patient #4, isolated ENF. B. Patient #5, not isolated ENF. Refer to Table 4 for details regarding the sites of primary disease and recurrences.

The leading cause of death among the patients was DM and the overall rate of DM was 36%. Of the 31 patients with local regional failure, 26 (22% of patients overall) had IFF. The other 5 patients (4.3% of all patients) developed ENF (Tables 3, 4 and Figure 2). Two of the 5 patients (1.7% of the overall group) developed isolated ENF as the first site of failure, whereas the other 3 patients developed in-field recurrence followed by out-of-field recurrence (any ENF). All of the elective nodal failures occurred in regions that did not receive a therapeutic dose of incidental radiation (> 45 Gy, Table 4). The Representative cases of ENF were shown in Figure 2. The locations of recurrence were mixed and not clearly predictable on the basis of the location of the primary tumor. For all 88 patients with stage IIIA/B, the frequency of IFF, ENF, isolated ENF, and DM were 23 (26%), 3 (3.4%), 1 (1.1%), and 36 (40.9%), respectively. Of the 27 patients with early-stage node-negative disease (stage IA/IB), 3 had IFF (11.1%), 1 had ENF (3.7%), 0 had isolated ENF (0%), and 5 had DM (18.5%). All 5 patients with ENF underwent PET for staging, and 1 of these patients was alive at the last follow-up (at 44 months).

**Table 3:** Disease recurrence pattern.

	N (%)
First site of failure (56 failures among 115 patients)	
Isolated Elective nodal failure	2 (1.7)
In-field failure	18 (15.7)
Distant metastasis	36 (31.3)
Any Elective nodal failures (isolated and non-isolated)	5 (4.3)
Stage I/II	2
Stage III	3

**Table 4:** Patient characteristics for patients with any failures in elective nodal regions

Patient with ENF	T-Stage	N-Stage	Pathologic diagnosis	Primary tumor location	Dose (Gy)	Chemotherapay	Site of failure	Dose to failure site (Gy)
1	2	1	Squamous	Left hilum	60	None	Right supraclavicular*	< 1.0
2	4	3	Adenocarcinoma	Left lower lobe	63	Concurrent carboplatin/paclitaxel	Ipsilateral mediastinum	22.5
3	2/1	0/0	Adenocarcinoma	Left lower lobe/right upper lobe†	66	None	Left hilum, Left supraclavicular, Right supraclavicular	23.3, 24.4, 44.3
4	2	3	Adenocarcinoma	Right upper lobe	60	Induction and concurrent carboplatin/paclitaxel	Right supraclavicular*	10.7
5	2	3	Squamous	Right upper lobe, contralateral mediastinum, right hilum	63	Concurrent carboplatin/paclitaxel	Left hilum	30.4

* Site of first failure in isolated elective nodal region.

† Synchronous primary tumor

## Discussion

Using PET for staging and treatment design, we found that definitive 3-D radiotherapy without ENI was associated with less than 2% isolated ENF in our study. IFRT alone appears adequate for inoperable NSCLC. The main rationale for foregoing ENI is that gross disease is not being controlled according to the high local recurrence rates (22%) within the previously irradiated tumor volume and the high rates of DM (36%). If gross disease cannot be controlled, then enlarging the irradiated volumes to include areas that might harbor microscopic disease seems unnecessary. Three major factors have changed since RTOG 73-01 established standard irradiation doses and volumes (including ENI): the advent of CT-based 3D-CRT, the use of chemotherapy, and better staging and target delineation using PET.

Emerging clinical data show that omitting prophylactic lymph node irradiation does not reduce the local control rate for patients receiving definitive radiotherapy, with isolated ENF rates of 0% to 8% [5]. Rosenzweig and colleagues recently published results for a series of 524 patients treated definitively with 3D-CRT targeted to involved-field volumes without ENI [7]. Only 6.1% of patients suffered isolated ENF, while 49% developed IFF. Belderbos et al also reported a 3% isolated ENF rate in patients with PET-staged NSCLC receiving dose-escalated radiotherapy without ENI [8]. Senan and colleagues reported no ENF in patients with stage III disease treated with 70 Gy of radiation without ENI [9]. A recently published randomized trial also supported the benefit of IFRT, with improved overall survival using dose-escalation in the IFRT arm but not in ENI arm [10]. The multicenter study RTOG 9311 also showed that only 6.7% patients developed isolated ENF in NSCLC treated with dose-escalated 3D-CRT without ENI [11].

The explanation for these lower-than-expected ENF rates could be twofold. First, incidental doses to the ipsilateral hilum, paratracheal, and subcarinal nodes approach 40 to 50 Gy when these regions are not intentionally irradiated [12]. Second, lung cancer patients have multiple competing causes of mortality, including cancer and underlying comorbid illnesses. Patients may die of local failure, distant failure, or intercurrent illness without detection of ENF. Therefore, considering the high percentages of IFF/DM and the short duration of overall survival using the current dose (60 to 70 Gy) and technique (3D-CRT) of therapy, the very low rate of isolated ENF justifies the use of IFRT alone in NSCLC.

PET scanning used for staging in our study may explain our very low rate (1.7%) of isolated ENF compared with other studies that did not use PET scans [5]. In addition, our recent published data has shown that tumor size and PET standardized uptake value (SUV) predicted the chance of IFF accurately [13]. In NSCLC, the chance of IFF was 100% when the SUV for lymph node(s) was above 13.8 compared with 15% when the SUV was less than or equal to 13.8 [13]. We found the SUV of the primary tumor to have the same predictive value. Thus, in patients with NSCLC, it is important to deliver adequate doses of radiation to the involved nodal or mediastinal areas, particularly those areas with high SUVs. Adjusting the dose to deliver higher levels of radiation to high-SUV areas seems to be indicated.

Efforts to improve local control in patients treated with radiation therapy for NSCLC have led to the development of dose escalation strategies and concurrent chemoradiotherapy. However, significant toxicities are associated with dose escalation and concurrent CHT and, therefore, ENI needs to be minimized [14]. However, evolution of the increasingly conformal treatment approaches such as IMRT and proton therapy may reduce incidental doses to the elective nodal region [15]. The data from our undergoing clinical trials using IMRT or proton therapy in stage III NSCLC will provide valuable information about the impact of incidental doses on ENF. In addition, as we improve our clinical outcomes (with lower rates of IFF and longer survival) with dose escalated radiotherapy and concurrent CHT, we should re-analyze the ENF data. As patients survive longer and IFF is reduced, the microscopic disease in the elective nodal region may become a greater issue.

In our study, the low rates of isolated ENF were not a consequence of unusually high rates of IFF, which can reduce the observed rate of isolated ENF. The 1- and 2-year local control rates of 83% and 60% were comparable to those previously reported [11]. The pattern of ENF does not suggest any needed modifications to the treatment fields. The ENFs occurred in patients of diverse stages, treatment approaches, and tumor locations. Only 2 of the 5 patients with ENF developed recurrences in next order draining lymphatics. Furthermore, of the 5 ENFs, 3 occurred after IFF and may represent the spread of in-field recurrences rather than true ENF.

In this study, the target volume was generally restricted to involved regions, with some of the first-echelon nodes treated if they were visible and adjacent to gross disease and if coverage of these nodes would not change normal tissue toxicity significantly, as decided on an individual basis by the radiation oncologist. This approach was supported by the observation that the highest rates of micrometastatic disease in mediastinal lymph nodes occurred nearest to the tumor, mainly in peribronchial and hilar locations [16]. Many factors are currently considered in the decision to include first-echelon nodes, such as imaging characteristics of the lymph nodes, proximity to the primary tumor, and the ability to meet dose constraints. Recently, endobronchial ultrasound-guided transbronchial needle aspiration (EBUS-TBNA) has become widely used to stage mediastinal lymph nodes. Dr. Herth et al [17] reported that EBUS-TBNA detected lymph node involvement in 9/97 patients with clinical stage I NSCLC with a normal CT image and negative PET of lymph node areas. EBUS-TBNA may be considered to verify the lymph node involvement for suspicious lymph nodes in GTV delineation. This procedure may further improve the accuracy of lymph node staging and target delineation. It should be noted, however, that the sensitivity of EBUS-TBNA is about 90% and common lymph node stations sampled are level 2, 4, 7, 10 and 11 but not level 5, 6, 8 and 9.

## Conclusion

In the current era of PET-guided radiation treatment planning and 3D-CRT, restricting the target volume to involved nodal regions results in minimal (< 2%) isolated ENF. ENI does not appear to be indicated for unresectable NSCLC. Future strategies should continue to be directed at improving in-field control and preventing distant metastatic disease.

## Abbreviations

GTV: gross tumor volume; CTV: Clinical target volume; PTV: Planning target volume; IFF: in-field failure; ENF: elective nodal failure; ENI: elective nodal irradiation; DM: distant metastasis; CHT: chemotherapy; 3-DCRT: 3-dimensional radiation therapy; IMRT: intensity modulated radiation therapy.

## Competing interests

The authors declare that they have no competing interests.

## Authors' contributions

ES participated in research design, coded the patient database, conducted the analysis and wrote the manuscript draft. JYC designed the project, analyzed the data and revised the manuscript. AK helped with the database and data analysis. JDC and RK provided additional guidance and support for this research.

## References

[B1] Perez C, Stanley K, Grundy G, Grundy G, Hanson W, Rubin P, Kramer S, Brady LW, Marks JE, Perez-Tamayo R, Brown GS, Concannon JP, Rotman M (1982). Impact of irradiation technique and tumor extent in tumor control and survival of patients with unresectable non-oat cell carcinoma of the lung: report by the Radiation Therapy Oncology Group. Cancer.

[B2] Bradley J, Thorstad W, Mutic S, Miller TR, Dehdashti F, Siegel BA, Bosch W, Bertrand RJ (2004). Impact of FDG-PET on radiation therapy volume delineation in non-small-cell lung cancer. Int J Radiat Oncol Biol Phys.

[B3] Schild SE (2008). Elective nodal irradiation (ENI) doesn't appear to provide a clear benefit for patients with unresectable non-small cell lung cancer (NSCLC). Int J Radiat Oncol Biol Phys.

[B4] Kong FM, Gaspar LE, Komaki R, Sun A, Bonner JA, Choy H, Wang L, Morris D, Sandler HM, Movsas B (2007). Patterns of practice in radiation dose prescription and treatment planning for patients with lung cancer among members of American Society of Therapeutic Radiology and Oncology. Int J Radiat Oncol Biol Phys.

[B5] Belderbos JSA, Kepka L, Kong F, Martel MK, Videtic GMM, Jeremic B (2008). Report from the international atomic energy agency (IAEA) consultants' meeting on elective nodal irradiation in lung cancer: non-small cell lung cancer (NSCLC). Int J Radiat Oncol Biol Phys.

[B6] Therasse P, Arbuck SG, Eisenhauer EA, Wanders J, Kaplan RS, Rubinstein L, Verweij J, Van Glabbeke M, van Oosterom AT, Christian MC, Gwyther SG (2000). New guidelines to evaluate the response to treatment in solid tumors. European Organization for Research and Treatment of Cancer, National Cancer Institute of the United States, National Cancer Institute of Canada. J Natl Cancer Inst.

[B7] Rosenzweig KE, Sura S, Jackson A, Yorke E (2007). Involved-field radiation therapy for inoperable non small-cell lung cancer. J Clin Oncol.

[B8] Belderbos JS, Jaeger K, Heemsbergen WD, Seppenwoolde Y, Baas P, Boersma LJ, Lebesque JV (2003). First results of a phase I/II dose escalation trial in non-small cell lung cancer using three-dimensional conformal radiotherapy. Radiother Oncol.

[B9] Senan S, Burgers J, Samson MJ, can Klaceren RJ, Oei SS, van Sornsen de Koste J, Voet PW, Lagerwaard FJ, Maarten van Haarst J, Aerts JG, van Meerbeeck JP (2002). Can electivenodal irradiation be omittd in Stage III non-small cell lung cancer? An analysis of recurrences in a phase II study of induction chemotherapy and involved-field radiotherapy. Int J Radiat Oncol Biol Phys.

[B10] Yuan S, Sun X, Li MH, Yu JM, Ren RM, Yu YH, Li JB, Kiu XQ, Wang RB, Li BS, Kong L, Yin Y (2007). A randomized study of involved field irradiation versus elective nodal irradiation in combination with concurrent chemotherapy for inperable stage III nonsmall cell lung cancer. AM J Clin Oncol.

[B11] Bradley J, Graham MV, Winter K, Purdy JA, Komaki R, Roa WH, Ryu JK, Bosch W, Emami B (2005). Toxicity and outcome results of RTOG 9311: a phase I-II dose-escalation study using three-dimensional conformal radiotherapy in patients with inoperable non-small-cell lung carcinoma. Int J Radiat Oncol Biol Phys.

[B12] Martel MK, Sahijdak WM, Hayman JA (1999). Incidental dose to clinically negative nodes from conformal treatment fields for nonsmall cell lung cancer. Int J Radiat Oncol Biol Phys.

[B13] Klopp AH, Chang JY, Tucker SL, Sulman E, Balter P, Liu H, Bucci MK, Macapinlac H, Komaki R, Cox JD (2007). Intrathoracic patterns of failure for non-small-cell lung cancer with position-emission tomography/computed tomography defined target delineation. Int J Radiat Oncol Biol Phy.

[B14] Chang DT, Zlotecki RA, Olivier KR (2005). Re-examining the role of elective nodal irradiation: finding ways to maximize the therapeutic ratio. Am J Clin Oncol.

[B15] Chang JY, Liu HH, Komaki R (2005). Intensity modulated radiation therapy and proton radiotherapy for non-small cell lung cancer. Curr Oncol Rep.

[B16] Chen ZL, Perez S, Holmes EC, Wang HJ, Coulson WF, Wen DR, Cochran AJ (1993). Frequency and distribution of occult micrometastases in lymph nodes of patients with non-small-cell lung carcinoma. J Natl Cancer Inst.

[B17] Herth FJ, Eberhardt R, Krasnik M, Ernst A (2008). Endobronchial ultrasound-guided transbronchial needle aspiration of lymph nodes in the radiologically and positron emission tomography-normal mediastinum in patients with lung cancer. Chest.

